# Dose rate versus gantry speed performance evaluation for slow gantry speeds using DICOM RT plans

**DOI:** 10.1002/acm2.13786

**Published:** 2022-09-19

**Authors:** Alexander Z. Bredikin, Michael J. Walsh

**Affiliations:** ^1^ Department of Radiation Oncology MD Anderson Cancer Center at Cooper Voorhees New Jersey USA

**Keywords:** dose rate, gantry speed, linac, quality assurance, VMAT

## Abstract

**Purpose:**

Varian provides a DICOM RT Plan file that users can deliver to the electronic portal imaging device (EPID) panel to confirm the linear accelerator delivers consistent dose output across several regions of interest for varying dose rates and gantry speeds (DRGS). This work investigates if (a) the vendor‐provided DRGS DICOM RT Plan is valid within the gantry speed range of stereotactic body radiation therapy (SBRT) treatments, and (b) if output constancy is maintained at those gantry speeds on a TrueBeam.

**Methods:**

Python code was written to iterate through all control points in the DICOM RT Plan files for 21 SBRT patients and the MU per degree values were calculated for each control point. A histogram was generated to illustrate how MU per degree was distributed among the control points from the patient plans. Then, the total number of MUs was increased in the vendor‐provided DRGS DICOM file to make a “modified DRGS DICOM RT Plan,” which surpasses the maximum MU per degree value found in the patient plans, forcing the gantry to travel at slow speeds and deliver more MU per degree over the same arc length (representative of those during SBRT treatment delivery). The modified DRGS DICOM RT Plan file was then delivered on a TrueBeam to acquire EPID images of the dose distribution. The EPID images were analyzed with Pylinac, a Python library that analyzes DICOM RT images acquired during routine linac QA.

**Results:**

Over 83% of patient DICOM RT Plan control points had MU per degree values greater than the MU per degree values in the vendor‐provided DRGS DICOM file. The Pylinac analysis of the EPID‐acquired images found a maximum deviation of 0.4% from machine baselines.

**Conclusions:**

The modified DRGS DICOM file can be used to determine if a TrueBeam linac is operating within specifications even when very low gantry speeds are reached.

## 1 INTRODUCTION

A seminal 2008 article from Otto invited the clinical introduction of volumetric‐modulated arc therapy (VMAT), where beam delivery is characterized by rotational arcs and the simultaneous modulation of gantry speed, dose rate, and multi‐leaf collimator (MLC) leaf position. This permits efficient treatment delivery with high‐dose conformity to the target.^1^

Ling et al. were among the first to detail commissioning and quality assurance tests for VMAT‐enabled linear accelerators (linac). One test they proposed was designed to deliver uniform dose over seven different regions of the field (using MLC‐defined “strips”), and each region uses a unique combination of dose rate and gantry speed. This tests the ability of the linac to modulate dose rate and gantry speed (DRGS) and confirm that the machine is able to deliver uniform output for a variety of delivery conditions.^2^

Varian (Varian Medical Systems, Palo Alto, CA, USA) provides DICOM RT Plan files corresponding to the recommended tests from Ling et al.^3^ One file, T2_DoseRateGantrySpeed_M120.dcm, is used to carry out the DRGS test. This work investigates if (a) the vendor‐provided DRGS DICOM RT Plan reaches monitor unit (MU) per degree values relevant to stereotactic body radiation therapy (SBRT) treatments, and (b) if output constancy is maintained at those MU per degree values on a TrueBeam.

MU per degree is used as a proxy for gantry speed in this work. Once the linac reaches a “critical” MU per degree value, dependent on the linac's maximum gantry speed (deg/s)_max_ and dose rate (MU/min)_max_, it will maximize dose rate and modulate gantry speed. As such, gantry speed will decrease as MU per degree increases beyond the critical MU per degree value. The critical MU per degree value is calculated using

(1) $${\left(\frac{\mathrm{MU}}{\deg}\right)}_{\mathrm{critical}}=\frac{{\left(\frac{\mathrm{MU}}{\min}\right)}_{\max}}{\left(60\right){\left(\frac{\deg}{s}\right)}_{\max}}$$

## 2 METHODS

### 2.1 Exploration of representative data

The current range of MU per degree values that Varian's DRGS DICOM RT Plan file tests was first determined. To do this, Python (version 3.9.7) code was written using the Anaconda command line client (version 1.9.0) to iterate through all control points in a given DICOM RT Plan file. A control point (CP) is composed of a gantry angle, cumulative fractional MU, and MLC leaf positions. MU per degree was derived using MU per control point (MU/CP) and degrees per control point (deg/CP) values obtained from the DICOM RT Plan file:

(2) $$\frac{\mathrm{MU}}{\deg}=\frac{\mathrm{MU}/\mathrm{CP}}{\deg/\mathrm{CP}}$$

These data were stored using the Pandas library (version 1.0.5) in a dataframe for further analysis.

The overlap between the current range of MU per degree values from the Varian DRGS DICOM RT Plan file and SBRT patient DICOM RT Plan files was investigated next to establish whether or not the Varian DRGS DICOM RT Plan file tests over the MU per degree values relevant to SBRT treatment plans. A representative sample of 21 recently treated SBRT patients was selected to determine the distribution of MU per degree value for SBRT plans. The breakdown of these patients is shown in Table 1.

**TABLE 1:** Stereotactic body radiation therapy treatment plans per site

**Treatment site**	**Number of patients**
Lung	12
Spine	3
Adrenal	2
Liver	2
Brain	1
Sacrum	1
Total	21

The range of MU per degree values in the SBRT patient plans was found to be greater than the MU per degree values in the vendor‐provided DRGS DICOM RT Plan file. A modified DRGS DICOM RT Plan file was created that surpasses the maximum MU per degree value found in the patient plans, thereby allowing the physicist to evaluate linac performance at slow gantry speeds (high MU per degree values). The Pydicom library (version 2.2.2) was employed to make the modifications to the vendor‐provided DRGS DICOM RT Plan file.^4^ The total number of MUs in the vendor‐provided DRGS DICOM RT Plan file was increased 10‐fold. Increasing the total number of monitor units without changing any other attributes of the plan file requires that more MU per degree be delivered over the same arc length, which forces the gantry to rotate at slow speeds (representative of those during SBRT treatment delivery) to accomplish this.

### 2.2 DICOM RT image analysis

The modified DRGS DICOM RT Plan file was then delivered on a Varian TrueBeam linac. A dosimetry imaging procedure was added to the treatment fields so that the electronic portal imaging device (EPID) acquired signal while the MLC‐defined strips and open field beams were being delivered. The TrueBeam used in this study is equipped Varian's Digital Megavolt Imager (DMI‐0105), whose performance characteristics have been described elsewhere in the literature.^5^ Analysis of these images was performed using Pylinac (version 3.0.1), a Python library that analyzes DICOM RT Images acquired during routine linac QA.^6^ The MLC strip image is normalized against the open field image to create a “corrected” image. The deviation for one of the MLC‐defined regions is calculated by the ratio of the average signal readings in that segment to the overall average signal within all of the MLC‐defined strips:

(3) $$\begin{array}{ccc} & & \mathrm{Deviation}\;\mathrm{for}\;\mathrm{segment}\;i\\ & & \;=\;\left(\frac{\mathrm{Average}\;\mathrm{corrected}\;\mathrm{reading}\;\mathrm{for}\;\mathrm{segment}\;i}{\mathrm{Averaged}\;\mathrm{corrected}\;\mathrm{reading}\;\mathrm{of}\;\mathrm{all}\;\mathrm{segments}}\times 100\right)-100 \end{array}$$

## 3 RESULTS

### 3.1 Exploration of representative data

**FIGURE 1:**
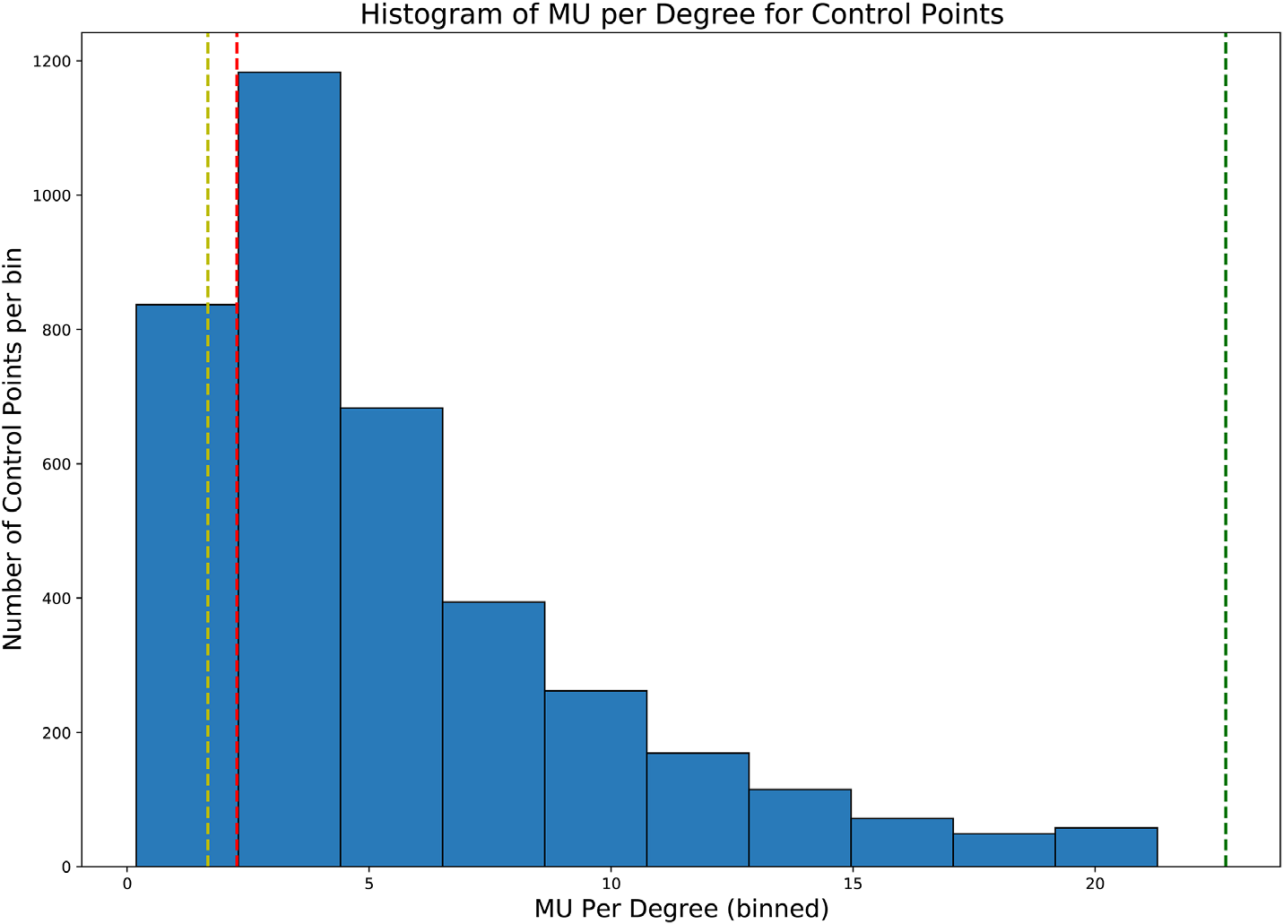
Distribution of monitor unit (MU) per degree for stereotactic body radiation therapy (SBRT) treatment plans. The dashed yellow line shows the “critical” MU per degree value (1.67 MU/deg) where, once exceeded, the dose rate is maximized and the gantry speed is modulated. The dashed red line on the histogram represents the maximum MU per degree value tested using the vendor‐provided DRGS DICOM RT Plan file. The dashed green line indicates maximum MU per degree values in the modified DRGS DICOM RT Plan file.

The histogram in Figure 1 illustrates how MU per degree was distributed among the control points from the SBRT patient plans. Total 3822 control points were analyzed. The dashed yellow line shows the critical MU per degree value (1.67 MU/deg), where, once exceeded, the dose rate is maximized and the gantry speed is modulated. The critical MU per degree value is calculated using Equation (1).

The dashed red line in Figure 1 represents the maximum MU per degree value tested using the vendor‐provided DRGS DICOM RT Plan file. Over 83% of the control points from the clinical SBRT plans evaluated in this study had MU per degree values exceeding the highest MU per degree value in the vendor‐provided DRGS DICOM RT Plan file.

The maximum MU per degree value from the patient plans was found to be 21.29 MU/deg. Per documentation from Varian, the TrueBeam linac is able to deliver a maximum dose rate of 60 MU/deg.^7^ The number of monitor units in the vendor‐provided DRGS DICOM RT Plan file had to be increased 10‐fold (from 250 to 2500 MU) in order to accommodate an MU per degree value exceeding the maximum from the patient plans. In doing so, the modified DRGS DICOM RT Plan delivers a maximum of 22.72 MU/deg. This is demarcated by the dashed green line in Figure 1.

### 3.2 DICOM RT image analysis

The MLC strip and open field images acquired with the EPID panel were analyzed with Pylinac. The Pylinac analysis found a maximum deviation of 0.4% when comparing the current baselines from the vendor‐provided DRGS file to the modified file. The results are shown in Table 2. Current baselines were determined using 27 months of historical data, and analysis of the images in this study was identical to the analysis of the historical data.

**TABLE 2:** Results of Pylinac analysis

ROI #	Measured mean values	Baseline	Difference
ROI 1	1.01	1.012	−0.2%
ROI 2	1.001	1.004	−0.3%
ROI 3	1.0	1.002	−0.2%
ROI 4	0.996	0.992	0.4%
ROI 5	0.994	0.995	−0.1%
ROI 6	0.998	0.998	0.0%
ROI 7	1.0	0.997	0.3%

## 4 DISCUSSION

Based on these data, the vendor‐provided DRGS DICOM RT Plan file does not evaluate the entire clinically relevant range of MU per degree, but the modified DRGS DICOM RT Plan file does. In their customer acceptance documents, Varian states that machines using the DRGS DICOM RT Plan they provide typically achieve deviation values (calculated using Equation 3) below 3% for any given ROI.^3^ The results of this study are within that value, evidencing that the linac continues to operate as expected even when gantry speeds are low and MU per degree values are high (which may be the case in SBRT treatments).

The TrueBeam linacs used in this study do not have flattening‐filter‐free (FFF) beams commissioned, so their maximum nominal dose rate is 600 MU/min at isocenter. Higher nominal dose rates, like those offered by FFF beams, would increase the critical MU per degree value.

## 5 CONCLUSIONS

This study suggests that a modified version of the vendor‐provided dose rate versus gantry speed DICOM RT Plan file can be used to determine if a TrueBeam linac is operating within specifications, even when very slow gantry speeds are reached. DICOM and Python scripting were used as part of this investigation, in hopes that other clinics are able to adopt the methodology presented in this work.

## AUTHOR CONTRIBUTIONS

Alexander Z. Bredikin made substantial contributions on the design of the work and on the acquisition, analysis, and interpretation of data for this work. Michael J. Walsh made substantial contributions on conception of the work and on interpretation of data for this work. Both the authors contributed in the drafting and revision of this work for important intellectual content. The authors give approval of the version to be published. The authors agree to be held accountable for all aspects of the work in ensuring that questions related to the accuracy or integrity of any part of the work are appropriately investigated and resolved.

## CONFLICT OF INTEREST

The authors have no conflicts of interest related to this work.

## Data Availability

The data and scripts that support the findings of this study are available from the corresponding author upon reasonable request.

## References

[1] Otto K . Volumetric modulated arc therapy: IMRT in a single gantry arc. Med Phys. 2008;35(1):310‐317. doi: 10.1118/1.2818738 18293586 10.1118/1.2818738

[2] Ling CC , Zhang P , Archambault Y , Bocanek J , Tang G , LoSasso T . Commissioning and quality assurance of RapidArc radiotherapy delivery system. Int J Radiat Oncol Biol Phys. 2008;72(2):575‐581. doi: 10.1016/j.ijrobp.2008.05.060 18793960 10.1016/j.ijrobp.2008.05.060

[3] Varian Medical Systems . RapidArc QA Test Procedures for TrueBeam . Varian Medical Systems; 2014.

[4] Darcy Mason, Scaramallion, Mrbean‐Bremen, et al. Pydicom/Pydicom: Pydicom 2.2.2. Zenodo; published October 1, 2021. doi: 10.5281/zenodo.5543955

[5] Miri N , Keller P , Zwan BJ , Greer P . EPID‐based dosimetry to verify IMRT planar dose distribution for the aS1200 EPID and FFF beams. J Appl Clin Med Phys. 2016;17(6):292‐304. doi: 10.1120/jacmp.v17i6.6336 27929502 10.1120/jacmp.v17i6.6336 PMC5690494

[6] Kerns JR , Taylor R . Pylinac Release 3.0 . Pylinac. Accessed August 4, 2021 2021. https://pylinac.readthedocs.io/en/latest/index.html

[7] Varian Medical Systems . TrueBeam Administrators Guide . Varian Medical Systems; 2019.

